# *PCSK6* VNTR Polymorphism Is Associated with Degree of Handedness but Not Direction of Handedness

**DOI:** 10.1371/journal.pone.0067251

**Published:** 2013-06-27

**Authors:** Larissa Arning, Sebastian Ocklenburg, Stefanie Schulz, Vanessa Ness, Wanda M. Gerding, Jan G. Hengstler, Michael Falkenstein, Jörg T. Epplen, Onur Güntürkün, Christian Beste

**Affiliations:** 1 Department of Human Genetics, Ruhr-University, Bochum, Germany; 2 Institute of Cognitive Neuroscience, Biopsychology, Department of Psychology, Ruhr University, Bochum, Germany; 3 Leibniz Research Centre for Working Environment and Human Factors (IfADo), Dortmund, Germany; Nathan Kline Institute and New York University School of Medicine, United States of America

## Abstract

Although the left and right human cerebral hemispheres differ both functionally and anatomically, the mechanisms that underlie the establishment of these hemispheric specializations, as well as their physiological and behavioral implications, remain largely unknown. Since cerebral asymmetry is strongly correlated with handedness, and handedness is assumed to be influenced by a number of genetic and environmental factors, we performed an association study of *LRRTM1* rs6733871 and a number of polymorphisms in *PCSK6* and different aspects of handedness assessed with the Edinburgh handedness inventory in a sample of unrelated healthy adults (n = 1113). An intronic 33bp variable-number tandem repeat (VNTR) polymorphism in *PCSK6* (rs10523972) shows a significant association (significance threshold: p<0.0025, adjusted for multiple comparisons) with a handedness category comparison (*P* = 0.0005) and degree of handedness (*P* = 0.001). These results provide further evidence for the role of *PCSK6* as candidate for involvement in the biological mechanisms that underlie the establishment of normal brain lateralization and thus handedness and support the assumption that the degree of handedness, instead the direction, may be the more appropriate indicator of cerebral organization.

## Introduction

Handedness is the most obvious manifestation of cerebral lateralization in humans, with more than 90% preferring to use their right hand [1]. In addition to the usually measured direction of this lateralization (left versus right), handedness is also characterized by its degree (consistent *vs*. inconsistent) [2], [3]. Handedness is a heritable trait, and many studies are consistent in assuming a polygenic model of handedness which means that a large number of genetic factors with a small additive effect may contribute to the trait variance [4]. Additive genetic effects were shown to account for about a quarter of the variance with the remainder accounted for by non-shared environmental influences [5]–[7]. Linkage analyses have identified a number of loci of interest including chromosome regions 2p12-q11, 10q26 and 12q21–23 [8]–[11]. Yet, to date, only few specific genes have been suggested as candidates influencing handedness: Francks *et al.* (2007) reported evidence for the involvement of the imprinted gene leucine-rich repeat transmembrane neuronal 1 (*LRRTM1*) on chromosome 2p12 in the development of schizophrenia/schizoaffective disorder and human handedness [12]. LRRTMs bind neurexins and act as control factors of presynaptic and postsynaptic glutamatergic differentiation [13]. In detail, Francks *et al.* (2007) report the association of a three-marker haplotype within an intron of *CTNNA2*, 137kb upstream of *LRRTM1*, with schizophrenia when inherited paternally [12]. Furthermore, this haplotype was also found to be paternally associated with handedness in a sample of reading-disabled sibships, implying the existence of specific imprinting effects on human brain [12], [14], [15]. The imprinting effects within *LRRTM1* and their association with schizophrenia could be supported by a study analysing a cohort of German patients. In addition to the haplotype specific markers, imprinting effects were also observed for the non-synonymous SNP rs6733871 that leads to an amino acid change (N330S) in LRRTM1. Furthermore, using the quantitative transmission disequilibrium test (QTDT), association between variation in *LRRTM1* rs6733871 and handedness in a well-characterized dyslexia sample of German descent was observed [16].

Recently, a genome-wide association study for a quantitative measure of relative handskill in individuals with reading disability (RD) revealed association with SNPs in *PCSK6* (proprotein convertase subtilisin/kexin type 6, also known as PACE4) which is known to have regulatory function during anterior CNS patterning and left-right axis formation [17], [18]. The strongest association with the quantitative measure of relative hand skill (peg-board task) was found for two intronic *PCSK6* SNPs, rs9806256 and rs11855415. RD individuals carrying the minor allele showed significantly greater relative right-hand skill as compared with the frequent wild-type allele. Replication in independent cohorts of individuals with RD revealed the same effect for the minor rs11855415 allele, while the same allele showed a significant trend towards reduced hand skill in the general population [17].

Here we report an association study of *LRRTM1* rs6733871 and a number of polymorphisms in *PCSK6* and different aspects of handedness assessed with the Edinburgh handedness inventory in a sample of 1113 unrelated healthy Caucasian adults. The results show a significant association with an intronic 33bp variable-number tandem repeat (VNTR) polymorphism in *PCSK6* (rs10523972) and handedness.

## Results

By using data from 1113 unrelated individuals, we tested the *LRRTM1* SNP rs6733871 and a number of SNPs in *PCSK6* for association with different aspects of handedness (Table 1). Each individual reported their hand preference (*i.e*., right, left or ambidextrous) in response to 10 questions (*e.g*., which hand do you prefer to use when: writing? throwing? using scissors?). All responses were converted to a laterality quotient (LQ) using the formula (R − L)/(R+L)×100. LQ scores thus ranged from −100 for strong left-handedness to +100 for strong right-handedness. Individuals were assigned to one of six categories of handedness: 1) consistent left (LQ = −100, 1.9%), 2) inconsistent left (LQ>−100 and LQ ≤ −50, 4.1%), 3) ambidextrous with a tendency towards left (LQ>−50 and LQ >0, 2.0%), 4) ambidextrous with a tendency towards right (LQ = 0 and LQ<+50, 4.0%),5) inconsistent right (LQ ≥ +50 and LQ<+100, 40.5%), or 6) consistent right (LQ = +100, 47.5%).

**Table 1:** p-values for the different association tests (the LQ has been analyzed using an ANOVA, while handedness groups, handedness direction and degree of handedness have been analyzed using Kruskal-Wallis-tests).

Gene	SNP	LQ	Handedness groups	Handedness direction	Degree of handedness
*LRRTM1*	rs6733871	0.35	0.34	0.54	0.41
*PCSK6*	rs11855415	0.33	0.84	0.13	0.88
	rs9806256	0.89	0.77	0.69	0.43
	rs1947942	0.37	0.21	0.41	0.33
	rs10523972	0.02	0.0005*	0.07	0.001*

Effects significant at the p<0.0025 level (the adjusted p value for multiple comparision) are given in bold.

Note: * = *p*<0.0025 (Bonferroni corrected p value).

The *p*-values for all genotype group comparisons for LQ, handedness groups (consistent left-handers, inconsistent left-handers, ambidexter with a tendency towards left-handedness, ambidexter with a tendency towards right-handedness, inconsistent right-handers, consistent right-handers), handedness direction (left, right) and degree of handedness (consistent, inconsistent).

When testing LQ, handedness direction (left, right), degree of handedness (consistent *vs.* inconsistent) and affiliation to one of the six categories of handedness for association with the *LRRTM1* and *PCSK6* one SNP rendered p-values below p<0.0025, the adjusted significance level for multiple comparisons (Table 1). The analyzed SNPs in *PCSK6* lie in a 12-kb region spanning introns 14–18 and are in low to high linkage disequilibrium (LD, D’ = 0.07–0.87) with each other. Rs10523972 is an intronic 33bp variable-number tandem repeat (VNTR) polymorphism in *PCSK6* with eight different alleles (4–11 copies); of which 6 and 9 copies were most frequently observed (20.4% and 70.2%). Dichotomizing these alleles into short (≤6 repeats) and long (≥9 repeats) alleles reveals significant associations for the handedness category comparison (Kruskal-Wallis test: χ^2^ = 15.07; p = 0.0005) and the degree of handedness (Kruskal-Wallis test: χ^2^ = 13.32; p = 0.001).

Moreover, the results show that among individuals heterozygous for the long and short *PCSK6* VNTR alleles the number of consistent right-handers was much lower (39.03%) than in individuals homozygous for the long allele (50.52%). The findings for individuals homozygous for the short allele should be interpreted carefully, since this group is very small (n = 55, Table 2).

**Table 2:** Observed frequencies of handedness in relation to the *PCSK6* rs10523972 alleles.

*PCSK6* VNTR alleles (n)	CLH	ILH	ALH	ARH	IRH	CRH
**long/long** (685)	13 (1.93%)	23 (3.42%)	11 (1.63%)	25 (3.71%)	261 (38.78%)	340 (50.52%)
**long/short** (339)	7 (1.99%)	21 (5.98%)	9 (2.56%)	17 (4.84%)	160 (45.58%)	137 (39.03%)
**short/short** (55)	1 (1.82%)	0 (0%)	1 (1.82%)	2 (3.64%)	21 (38.18%)	30 (54.55%)

Numbers and percentages of consistent left-handers (CLH), inconsistent left-handers (ILH), ambidexter with a tendency towards left-handedness (ALH), ambidexter with a tendency towards right-handedness (ARH), inconsistent right-handers (IRH) and consistent right-handers (CRH) for the combined *PCSK6* rs10523972 alleles.

The significant degree of handedness effect (Kruskal-Wallis test: χ^2^ = 13.32; p = 0.001) indicates a difference in the number of participants with consistent and non-consistent handedness. To further investigate this effect, we compared the distribution of consistent and non-consistent handedness for the different allele combinations against a uniform 50/50 distribution, resembling the overall distribution of consistent or inconsistent handed participants (consistent: 527 individuals, inconsistent: 552 individuals). This analysis revealed that heterozygous participants were more prone to inconsistent hand preference (consistent: 140; inconsistent: 199; χ2 = 10.27; *P* = 0.001, Table 1). Yet, the effect failed to reach significance for both homozygous allele combinations (long/long, consistent: 357; inconsistent: 328; χ2 = 1.23; p = 0.27 and short/short, consistent: 30; inconsistent: 25; χ2 = 0.46; p = 0.5).

Scerri et al. (2011) report a significant association with the minor allele of rs11855415 and increased relative right-hand skill in individuals with RD. Yet, the minor allele showed a significant trend towards reduced laterality of hand skill in the general population [17]. In order to compare the results we also z-standardized the LQ scores (mean = 0, SD = 1) and plotted their distribution for both rs118555415 and rs10523972 (see Fig. S1 and Fig. S2). The distribution of LQ scores for the minor alleles (rs118555415 AA and rs10523972 short/short), appear more tightly clustered around the mean than the frequent alleles, suggesting that carriers of the minor alleles show reduced variability in relative hand skill as compared to carriers of the frequent alleles. Further comparing the absolute value of the z-standardized LQ as a quantitative trait between these groups (see Fig. S3 and Fig. S4) indeed revealed that the homozygous carriers of the minor alleles for rs118555415 and rs10523972 showed the lowest absolute z-standardized scores (0.52 and 0.52) as compared with the homozygous carriers of the frequent alleles and the heterozygotes (0.60 and 0.61; 0.69 and 0.68). Yet, the effect for both SNPs failed to reach nominal significance (p = 0.14 and p = 0.22).

## Discussion

**Figure 1:**
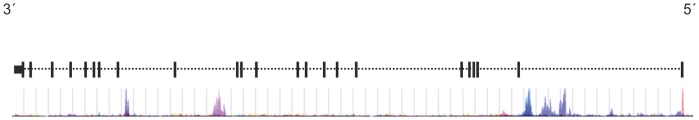
Graphical representation of the analyzed single-nucleotide polymorphisms in relation to the exon-intron structure at the 3’ end of *PCSK6.* H3K7Ac ChIP*-*Seq data from the ENCODE project, presented beneath, were obtained from the UCSC genome browser.

Our data show a statistical trend supporting earlier findings for an association of *PCSK6* with handedness. No supportive evidence was obtained for a possible association of the non-synonymous *LRTTM1* SNP rs6733871. However, neither the three-marker haplotype nor parental effects were analyzed. The finding that tandem repeat variation in *PCSK6* (rs10523972) shows a significant association with the degree of handedness could be explained by altered physiological and biological changes associated with this polymorphism. For example, VNTRs in regulating sequences can affect gene expression by altering the number of transcription factor binding sites or introducing changes in spacing between critical promoter elements [19]. Yet, gene expression can also be affected by modulating the activity of RNA-binding proteins or mediating effects on chromatin structure [19]. According to the Encyclopedia of DNA Elements (ENCODE) dataset, available for visualization and download via the UCSC Genome Browser (http://genome.ucsc.edu/), the region containing the *PCSK6* VNTR sequence clusters multiple transcription factor binding sites and is enriched for histone H3 lysine 27 acetylation (H3K27ac), a reliable chromatin marker of active (Fig. 1) [20], [21]. Additionally, the UCSC genome browser lists different spliced expressed sequence tags (ESTs) that emanate from the VNTR region, suggestive of a bidirectional promoter that can drive the expression of further truncated *PCSK6* mRNA variants that differ in their C- or N-terminal segments [22]. Thus, variation in its repeat number might well have considerable physical impact on the DNA, which can influence in a tissue-dependent manner gene regulation, DNA organization and many other mechanisms [23]. As already reviewed and discussed in detail in Scerri *et al.* (2011), PCSK6 plays an important role in the pathway for left-right asymmetry and has also been described to be a direct target of the transcription factor FOXP2, whose gene was the first one known to be involved in the development of speech and language [17], [24].

The lack of replication in our sample of the reported association with *LRRTM1* might be explained by different study designs, including the participant populations and definition of handedness. Individual differences in handedness were often reduced on the direction of hand preference, left versus right-handers. Yet, evidence has accumulated that the degree (consistent versus inconsistent) of hand preference probably reflects a more critical dimension on which the handedness groups differ [25], [26].

In summary, the results of our study provide evidence for an association of *PCSK6* variation and the degree of handedness in the general population. Together with previously published data on a *PCSK6* association and reduced variability in relative hand skill a healthy cohort [17] these results further support *PCSK6*s likely role as candidate for involvement in the biological mechanisms that underlie the establishment of normal brain lateralization and thus handedness. However, independent replication in larger cohorts is needed in order to further validate the present results.

Furthermore, our data support the assumption that the degree of handedness, *i.e.* how consistently a person prefers to use one versus the other hand over a wide variety of tasks may be the more appropriate indicator of cerebral organization. Future association studies should therefore take into consideration the different measures to define handedness. Understanding the complex genetic contribution to handedness could be crucial in order to advance our knowledge of normal brain functioning, but also the understanding of neurodevelopmental and psychiatric diseases.

## Methods

### Ethics Statement

The study was approved by the ethics committee, Ruhr-University Bochum. All participants gave written informed consent and were treated in accordance with the declaration of Helsinki.

### Study Participants

In total, 1113 genetically unrelated, healthy adult participants of Caucasian descent for at least two generations participated in the present study (655 women and 458 men). The participants had a mean age of 32.97 (SEM = 0.55) and none of them had a history of any neurological or psychiatric diseases. 895 of the participants were young adults (mostly university students), that were recruited for the present study, while the remaining 218 participants came from a sample of healthy elderly volunteers. All participants were native German speakers. No participants are included in the cohort that had been forced to write with the right hand in school, although they actually would have preferred to use the left.

### Handedness Assessment

Handedness was assessed using the Edinburgh handedness inventory [27]. In this questionnaire, participants have to indicate whether they prefer to use the left or right hand for ten different activities. Based on the participants answers, an individual laterality quotient (LQ) can be calculated using the formula LQ = [(R−L)/(R+L)]×100 (R = number of right sided preferences; L = number of left-sided preferences). The LQ has a range between +100 and −100. Positive values indicate right handedness and negative values left handedness, while higher absolute values indicate more consistent handedness and lower absolute values more inconsistent handedness or ambidexterity. Based on each participant’s individual LQ, three different measures were calculated in order to separately investigate different aspects of handedness that may be associated with different genetic variations. In order to gain a measure of handedness direction, participants were grouped into right-handers (RH; LQ between 0 and 100) and left-handers (LH; LQ between−100 and 0), an approach, that is commonly used in large-scale studies [6]. Additionally, we applied a more complex grouping method, classifying participants into six different handedness categories: consistent left handers (LQ = -100), inconsistent left-handers (LQ = -99 to −50), ambidexter with a tendency towards left-handedness (LQ = −49 to 0), ambidexter with a tendency towards right-handedness (LQ = 0 to 49), inconsistent right-handers (50 to 99) and consistent right handers (LQ = 100). Also, in order to gain a dichotomous measure of the degree of handedness independent of the individuals preferred hand, participants were grouped into individuals with consistent (LQ either 100 or −100) or individuals with inconsistent (all other LQ’s) handedness.

### Genotyping

For non-invasive sampling, exfoliated cells were brushed from the oral mucosa of the participants. DNA isolation was performed with QIAamp DNA mini Kit (50) (Qiagen GmbH, Hilden, Germany). SNPs were chosen for genotyping by selecting SNPs from the literature (*LRRTM1* rs6733871, *PCSK6* rs11855415, rs9806256) or from the UCSC genome browser, with particular attention to SNPs in conserved sequences (rs1947942) or SNPs that disrupt putative transcription factor binding sites (rs10523972). Genotyping of four SNPs (rs6733871, rs11855415, rs9806256 and rs1947942) was conducted by polymerase chain reaction (PCR) and differential enzymatic analysis with the PCR restriction fragment length polymorphism method. Genotyping of the *PCSK6* VNTR, rs10523972, was performed on the Beckman Coulter CEQ8000 8-capillary system using ’Fragment Analysis Module’ software (Beckman Coulter, Inc., Fullerton, USA). The genotypes were confirmed by sequence analysis. Oligonucleotides were designed using Primer Express 2.0 Software (Applied Biosystems). Further details of methodology and primer sequences are available upon request.

### Statistical Analysis

The statistical analyses were performed assuming a co-dominant effect for each polymorphism. Thus, all three genotypes for each polymorphism were analyzed separately. Since the LQ is an interval-scaled variable, it was analyzed parametrically using univariate analyses of variance (ANOVAs). Greenhouse-Geisser correction was applied when appropriate. Nominal-scaled variables (handedness categories, handedness direction and degree of handedness) were analyzed non-parametrically using the Kruskal-Wallis Test. In order to account for multiple comparisons, an effect was considered significant if the *p*-value was smaller than 0.05/20 (the overall number comparisons) = 0.0025 (Bonferroni correction), a highly conservative significance threshold. All statistics were calculated using IBM SPSS Statistics 20.

## Supporting Information

Figure S1 **Plot of z-standardized LQ score (mean = 0, SD = 1) distribution (y-axis) for each genotype of rs11855415 (x-axis) in 1113 genetically unrelated, healthy adult participants of Caucasian descent.**
(JPG)

Figure S2 **Plot of z-standardized LQ score (mean = 0, SD = 1) distribution (y-axis) for the dichotomized genotypes of rs10523972 (x-axis) in 1113 genetically unrelated, healthy adult participants of Caucasian descent.**
(JPG)

Figure S3 **Plot of the absolute value of the LQ score (y-axis) for each genotype of rs11855415 (x-axis) in 1113 genetically unrelated, healthy adult participants of Caucasian descent.**
(JPG)

Figure S4 **Plot of the absolute value of the LQ score (y-axis) for the dichotomized genotypes of rs10523972 (x-axis) in 1113 genetically unrelated, healthy adult participants of Caucasian descent.**
(JPG)
