# Supplementary figures and images for: PCSK6 VNTR Polymorphism Is Associated with Degree of Handedness but Not Direction of Handedness
Source: PLoS One. 2013 Jun 27;8(6):e67251. doi: 10.1371/journal.pone.0067251 (PMC3695088; doi:10.1371/journal.pone.0067251)

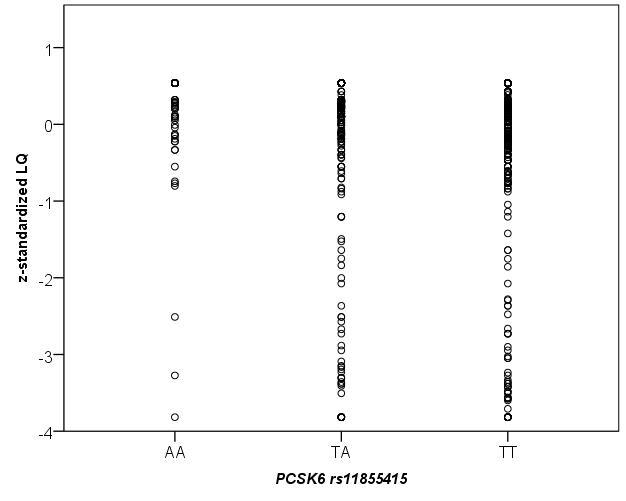

Supplement: Figure S1 — Plot of z-standardized LQ score (mean = 0, SD = 1) distribution (y-axis) for each genotype of rs11855415 (x-axis) in 1113 genetically unrelated, healthy adult participants of Caucasian descent. (JPG) [file pone.0067251.s001.jpg]

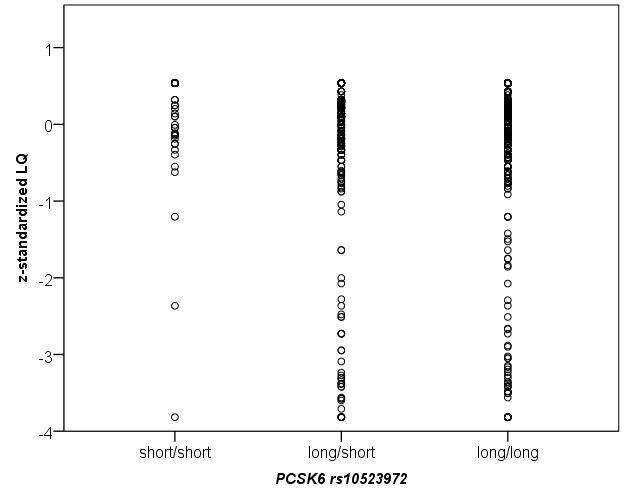

Supplement: Figure S2 — Plot of z-standardized LQ score (mean = 0, SD = 1) distribution (y-axis) for the dichotomized genotypes of rs10523972 (x-axis) in 1113 genetically unrelated, healthy adult participants of Caucasian descent. (JPG) [file pone.0067251.s002.jpg]

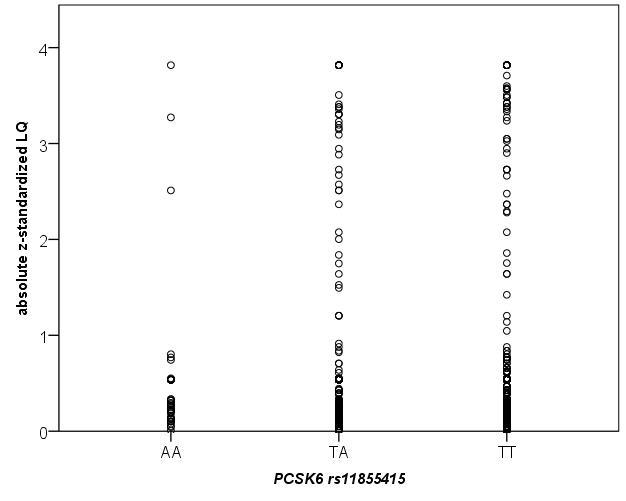

Supplement: Figure S3 — Plot of the absolute value of the LQ score (y-axis) for each genotype of rs11855415 (x-axis) in 1113 genetically unrelated, healthy adult participants of Caucasian descent. (JPG) [file pone.0067251.s003.jpg]

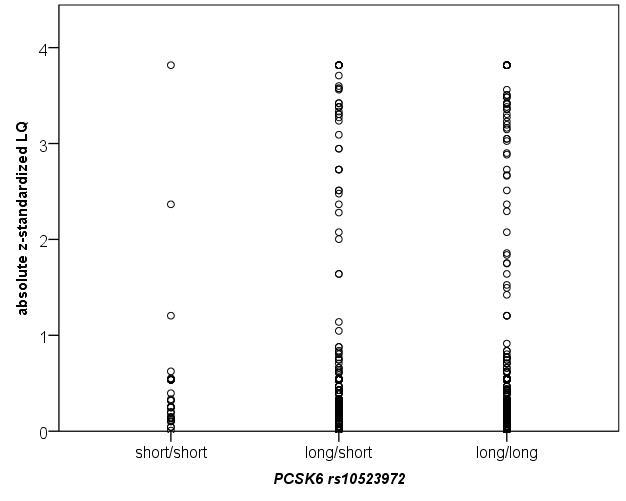

Supplement: Figure S4 — Plot of the absolute value of the LQ score (y-axis) for the dichotomized genotypes of rs10523972 (x-axis) in 1113 genetically unrelated, healthy adult participants of Caucasian descent. (JPG) [file pone.0067251.s004.jpg]
